# A Coiled-coil Clamp Controls Both Conformation and Clustering of Stromal Interaction Molecule 1 (STIM1)*

**DOI:** 10.1074/jbc.M114.610022

**Published:** 2014-10-23

**Authors:** Marc Fahrner, Martin Muik, Rainer Schindl, Carmen Butorac, Peter Stathopulos, Le Zheng, Isaac Jardin, Mitsuhiko Ikura, Christoph Romanin

**Affiliations:** From the ‡ Life Science Center JKU, Institute of Biophysics, Johannes Kepler University Linz, Gruberstrasse 40, 4020 Linz, Austria,; § Department of Physiology and Pharmacology, Western University, London, Ontario N6A 5C1, Canada, and; ¶ Princess Margaret Cancer Centre and Department of Medical Biophysics, University of Toronto, Toronto, Ontario M5G 1L7, Canada

**Keywords:** Calcium Release-activated Calcium Channel Protein 1 (ORAI1), Fluorescence, Patch Clamp, Signal Transduction, Stromal Interaction Molecule 1 (STIM1)

## Abstract

Store-operated Ca^2+^ entry, essential for the adaptive immunity, is initiated by the endoplasmic reticulum (ER) Ca^2+^ sensor STIM1. Ca^2+^ entry occurs through the plasma membrane resident Ca^2+^ channel Orai1 that directly interacts with the C-terminal STIM1 domain, named SOAR/CAD. Depletion of the ER Ca^2+^ store controls this STIM1/Orai1 interaction via transition to an extended STIM1 C-terminal conformation, exposure of the SOAR/CAD domain, and STIM1/Orai1 co-clustering. Here we developed a novel approach termed FRET-derived Interaction in a Restricted Environment (FIRE) in an attempt to dissect the interplay of coiled-coil (CC) interactions in controlling STIM1 quiescent as well as active conformation and cluster formation. We present evidence of a sequential activation mechanism in the STIM1 cytosolic domains where the interaction between CC1 and CC3 segment regulates both SOAR/CAD exposure and CC3-mediated higher-order oligomerization as well as cluster formation. These dual levels of STIM1 auto-inhibition provide efficient control over the coupling to and activation of Orai1 channels.

## Teaser

**Background:** STIM1 and Orai1 are key players in the store-operated Ca^2+^ entry.

**Results:** The activation state of STIM1 is precisely controlled by heteromeric interaction between coiled-coil domains.

**Conclusion:** A coiled-coil clamp provides control over STIM1 conformation and clustering.

**Significance:** Understanding of the STIM1 C-terminal switching mechanism is crucial for the control of Orai1 activation.

## Introduction

Store-operated calcium entry represents an essential signaling pathway for T-cell activation and mast cell degranulation. Its activation is triggered by various extracellular ligands that bind to receptors and initiate a release of Ca^2+^ from the endoplasmic reticulum (ER),^4^ known as store depletion (1). The drop in ER Ca^2+^ levels induces oligomerization of the ER located stromal interaction molecule 1 (STIM1) that translocates to ER-plasma membrane (PM) junctions and activates the PM-resident Ca^2+^ selective channel Orai1 (2–11). The formed STIM1 puncta co-cluster with accumulations of Orai1 triggering cellular Ca^2+^ hot spots (12, 13). Several studies on STIM1- and Orai1-knock-out mice as well as the severe combined immunodeficiency syndrome (SCID) associated with the Orai1 R91W mutant have demonstrated the impact of store-operated channels in physiological and pathophysiological functions of mast cells, T-lymphocytes, platelets, and smooth muscle cells (7, 14–17).

**FIGURE 1.**
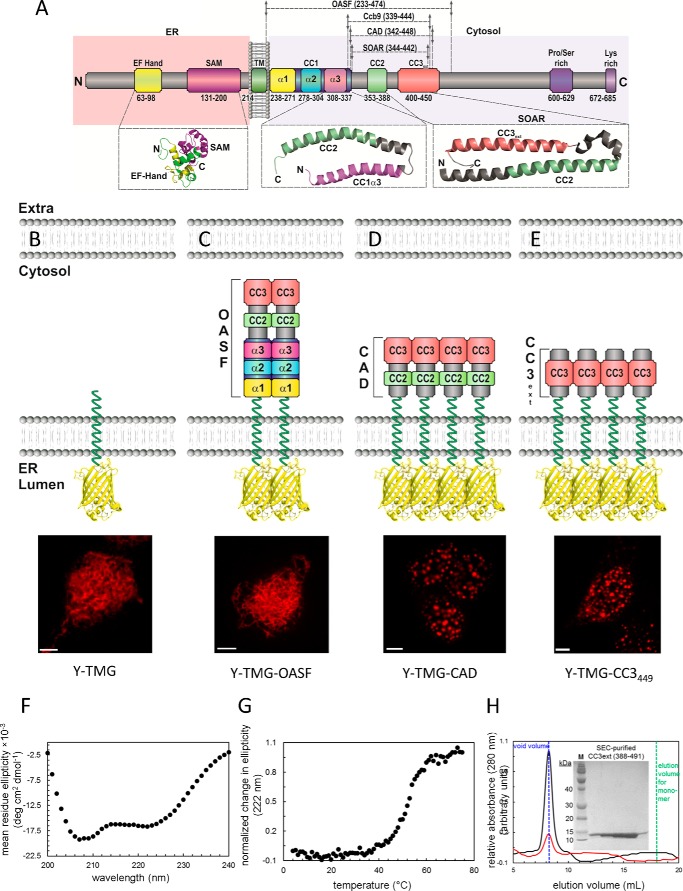
**Coiled-coil 1 controls formation of CAD clusters.** *A*, scheme of STIM1 depicting individual domains: ER luminal EF hand and SAM domain, transmembrane domain (*TM*), coiled-coil (*CC*) domains: CC1 subdivided into α1, α2, α3, respectively, CC2, CC3, Pro/Ser-rich domain, Lys-rich domain. *Numbers* correspond to amino acid positions of human STIM1. *Below*, solution NMR structures of STIM1 N-terminal domains as well as of the CC1_α3_-CC2 fragment, and the crystal structure of SOAR are magnified. *B–E*, FIRE system: schemes of FIRE constructs (Y-TMG-x; x: OASF, CAD, CC3_449_) used for fluorescence imaging experiments. Y (YFP) is located in the ER lumen, STIM1 fragments are located on the cytosolic side, attached via a Gly-linker to the ER transmembrane segment. All constructs were overexpressed in HEK 293 cells, and representative images are depicted: (*B*) Y-TMG (control) showed ER distribution without cluster formation. (*C*) Y-TMG-OASF resulted in ER distribution and no cluster formation. Y-TMG-CAD (*D*) and Y-TMG-CC3_449_ (*E*) revealed clear cluster formation. The bar in each fluorescence image corresponds to 5 μm. *F*, far-UV-CD spectrum of STIM1 CC3ext. The negative ellipticity minima at 208 and 222 nm are indicative of an α-helical fold. The spectrum was acquired at 0.25 mg ml^−1^ and is an average of 3 scans. *G*, thermal stability of STIM1 CC3ext. The sigmoidal unfolding profile suggests a cooperative unfolding and is consistent with the high α-helicity of CC3ext. The thermal melt was acquired at 0.25 mg ml^−1^. *H*, SEC of STIM1 CC3ext. After 100-μl injections of 1.25 mg ml^−1^ (*black trace*) and 0.25 mg ml^−1^ (*red trace*), CC3ext eluted in the void volume of the Superdex 200 10/30 GL column (*blue vertical broken line*) indicative of higher order oligomers with a molecular mass > 600 kDa. The expected elution volume of monomeric CC3ext is shown for reference (*green vertical broken line*). *Inset* depicts a Coomassie Blue-stained SDS-Page gel for fractions recovered following gel filtration showing monomeric CC3ext of 12.2 kDa.

STIM1 and Orai1 communicate via a direct interaction of their C-terminal strands (13, 18–20). In addition, STIM1 couples with a lower affinity to the N terminus of Orai1 (18, 19, 21). STIM1/Orai1 interaction is strictly controlled by store-depletion, via mechanistic steps including oligomerization and conformational rearrangement of STIM1 (22–25). The initial step upon ER [Ca^2+^] depletion is the di-/oligomerization of the luminal EF-hand and SAM domain of STIM1 (9, 10, 26). The cytosolic strand of STIM1 contains further di-/oligomerization domains (Fig. 1*A*). Specifically, the STIM1 Orai1 activation region, named SOAR (344–442) (27) or CAD (342–448) (18) plays a key role in STIM1-dependent activation of Orai1 channel. In the previously-reported crystal structure, it was found that the SOAR/CAD region is comprised of R-shaped coiled-coil 2 (CC2) and CC3 domains that further dimerize into a V-shaped structure (28). Despite of the compact structure observed in the crystalline state, the SOAR/CAD domain within full-length STIM1 is expected to reach the plasma membrane-embedded Orai1 channel via the long CC1 helix (28, 29). Several models have additionally attributed an inhibitory role to CC1 in as that STIM1-C terminus is kept in a tight, quiescent state because of an intramolecular interaction between an amphipathic α3 helix of CC1 (CC1_α3_) and CAD/SOAR (28, 30–32). Switching from a tight into an extended conformation is supposed to trigger CAD/SOAR exposure and STIM1 activation of Orai1 channels (30, 33, 34). Recently, we have revealed the atomic basis for the interaction of the C termini of Orai1 with the CC2 domains of the CAD/SOAR dimer which forms the STIM1-Orai1 association pocket (SOAP) (35). Our solution NMR data indicate that CC2 must undergo a conformational rearrangement relative to the CAD/SOAR crystal state to facilitate coupling to the Orai1 C-terminal domains in a manner that is congruent with the orientation revealed in the *Drosophila melanogaster* Orai structure (36). Further, we hypothesized that the CC3 domain of STIM1 provides homomerization function to arrange six STIM1 molecules in a higher-order oligomeric cluster within the Orai1 channel complex. However, it is not known, whether these conformational rearrangements indeed take place in live cells, and which additional, unidentified interactions they involve to tightly control both CAD/SOAR exposure and clustering in the STIM1 activation mechanism.

In this study we developed a novel live-cell imaging approach which enabled us to examine the proposed mechanistic steps in mammalian cells. The method detects FRET-derived interactions in a restricted environment (FIRE) for STIM1 that efficiently control cytosolic STIM1 conformational rearrangements including CAD/SOAR exposure, SOAP formation, and clustering. Using FIRE, we identified a new coiled-coil clamp in the heteromeric interaction between CC1_α1_ and CC3 helices that enables precise control over STIM1 C-terminal conformations. Further, we present a sequential, C-terminal switching mechanism providing dual levels of STIM1 auto-inhibition by linking CAD/SOAR exposure with cluster formation required for Orai1 channel coupling.

## EXPERIMENTAL PROCEDURES

### 

#### 

##### Molecular Cloning and Mutagenesis

Human ORAI1 (ORAI1; accession number NM_032790) was kindly provided by A. Rao's laboratory (Harvard Medical School). N-terminally tagged ORAI1 constructs were cloned via SalI and SmaI restriction sites of pECFP-C1 and pEYFP-C1 expression vectors (Clontech). Human STIM1 (STIM1; accession number NM_003156) N-terminally ECFP- and EYFP-tagged was kindly provided by T Meyer's Lab, Stanford University. For double-tagged STIM1 constructs, CFP was cloned into pEYFP-C2 via SacII and Xba1 and the OASF STIM1 fragment (233–474) was introduced via EcoRI and SacII. Double tagged OASF mutants (Δα1_238–271; Δα2_278–304; Δα3_308–337; Δα1α2_238–304; Δα2α3_278–337; L251S; R426L) were generated using the QuikChange XL site-directed mutagenesis kit (Stratagene). ECFP-STIM1 mutants (Δα1_238–271; Δα2_278–304; Δα3_308–337; Δα1α2_238–304; Δα2α3_278–337; L251S; R426L) were generated using the QuikChange XL site-directed mutagenesis kit (Stratagene). Constructs for the FIRE system consist of STIM1-signal peptide, EYFP (Y) or ECFP (C), 29 aa linker, STIM1 transmembrane domain, 32 glycine linker followed by protein fragment of interest (OASF 233–474; CAD 344–449; CC1 233–343; CC1_α1_ 233–276; CC1_α2_ 273–309; CC1_α3_ 303–342; CC2 344–399; CC3_420_ 388–420; CC3_430_ 388–430; CC3_449_ 388–449). Y- and C-TMG-CC1_α1_ L251S, Y- and C-TMG-CC3 R426L point mutants were generated using the QuikChange XL site-directed mutagenesis kit (Stratagene). The integrity of all resulting clones was confirmed by sequence analysis.

##### Confocal Microscopy

Confocal FRET microscopy was performed on HEK-293 cells, as previously described (48). In brief, a QLC100 Real-Time Confocal System (VisiTech Int.) connected to two Photometrics CoolSNAPHQ monochrome cameras (Roper Scientific) and a dual port adapter (dichroic: 505lp; cyan emission filter: 485/30; yellow emission filter: 535/50; Chroma Technology Corp.) was used for recording fluorescence images. This system was attached to an Axiovert 200M microscope (Zeiss, Germany) in conjunction with two diode lasers (445 nm, 515 nm) (Visitron Systems). Image acquisition and control of the confocal system was performed with a Visiview 2.1.1 software (Visitron Systems). Image correction due to cross-talk and cross-excitation were performed prior to the calculation. Therefore, appropriate cross-talk calibration factors were determined for each construct on every day of the FRET experiment. After threshold determination and background subtraction, the corrected FRET (E_app_) was calculated on a pixel-to-pixel basis with a custom-made software (37) integrated in MatLab 7.0.4 according to the method published in Ref. 38, with a microscope specific constant *G* value of 2.0. All experiments were performed at room temperature.

##### Recombinant Expression and Purification of STIM1 CC3ext

STIM1 CC3ext (*i.e.* residues 388–491) was subcloned into pET-28a using NheI and XhoI restriction sites. Protein was expressed in BL21 ΔE3 *Escherichia coli*, and Ni^2+^-nitrilotriacetic acid resin (Qiagen) was used to pull out the 6×His-tagged CC3ext from guanidinium-solubilized lysate. Refolding was performed by dilution in 20 mm Tris, 200 mm NaCl, 0.8 mm DTT, 0.8 mm EDTA, pH 8.5, and the protein was further purified by gel filtration after thrombin cleavage (10 units/mg overnight at 4 °C) of the 6×His affinity tag. Protein homogeneity was assessed by Coomassie-stained SDS-PAGE.

##### CD Spectroscopy

Far-UV-CD spectra were acquired between wavelengths of 240–200 nm using a Jasco J-815 CD spectrometer, equipped with a Peltier temperature controller (Jasco). Fixed-temperature data were collected using 0.1 cm pathlength cuvettes in 1 nm increments at a scan rate 20 nm min^−1^, 8-s averaging time, and 1-nm bandwidth. Thermal melts were acquired by monitoring the change in the 222 nm CD signal as a function of temperature (*i.e.* 4–75 °C) using 0.1-cm cuvettes, 8-s averaging time, 1-nm bandwidth, and 1 °C min^−1^ scan rate. Data were corrected for buffer contributions.

##### SEC with In-line Multi Angle Light Scattering (SEC-MALS)

SEC was performed on a Superdex S200 10/300 GL column linked to an AKTA FPLC system (GE Healthcare) at 4 °C. MALS measurements were performed in-line with the SEC using a three-angle (*i.e.* 45°, 90° and 135°) miniDawn light-scattering instrument equipped with a 690 nm laser (Wyatt Technologies); however, due to elution of the CC3ext in the void volume of the column, accurate molecular weight estimates could not be derived.

##### Whole Cell Patch Clamp Experiments

HEK-293 cells were transfected (Transfectin, Bio-Rad) with 1 μg of DNA of YFP-Orai1 and mCh-STIM1 constructs. Electrophysiological experiments were performed after 24 to 34 h, using the patch-clamp technique in whole-cell recording configurations at 21–25 °C. An Ag/AgCl electrode was used as reference electrode. Voltage ramps were applied every 5 s from a holding potential of 0 mV, covering a range of −90 to 90 mV over 1 s. For passive store depletion the internal pipette solution included (in mm): 145 Cs methane sulfonate, 20 EGTA, 10 HEPES, 8 NaCl, 3.5 MgCl_2_, pH 7.2. Standard extracellular solution consisted of (in mm) 145 NaCl, 10 HEPES, 10 CaCl_2_, 10 glucose, 5 CsCl, 1 MgCl_2_, pH 7.4. A liquid junction potential correction of +12 mV was applied, resulting from a Cl^−^-based bath solution and a sulfonate-based pipette solution. All currents were leak subtracted either by subtracting the initial voltage ramps obtained shortly following break-in with no visible current activation, or with constitutively active currents after 10 μm La^3+^ application at the end of the experiment.

## RESULTS

### 

#### 

##### Coiled-coil 1 Controls Formation of CAD Clusters

**FIGURE 2.**
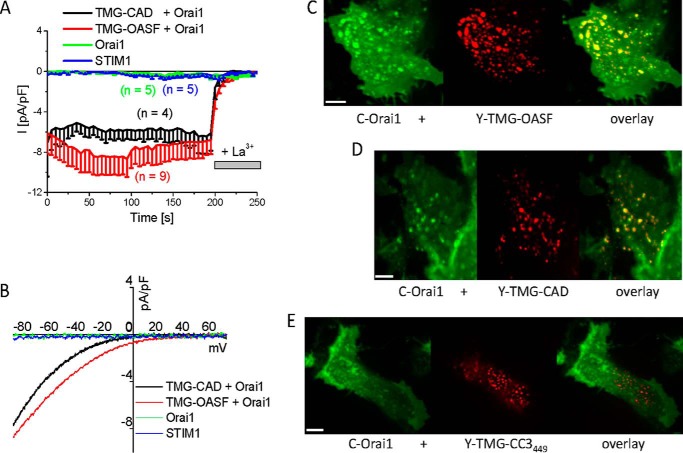
**ER-anchored CAD or OASF co-localizes with and constitutively activates Orai1 channels.** *A*, time course of inward currents from HEK293 cells co-expressing fluorescently labeled TMG-CAD + Orai1 and TMG-OASF + Orai1, as well as STIM1 or Orai1 as control, and (*B*) the respective current-voltage relationship. Fluorescence images showing localization and overlay of (*C*) C-Orai1 + Y-TMG-OASF, (*D*) C-Orai1 + Y-TMG-CAD, and (*E*) C-Orai1 + Y-TMG-CC3_449_. The bar in each fluorescence image corresponds to 5 μm.

Activation of the ER membrane-anchored STIM1 transpires through several mechanistic steps involving luminal di-/oligo-merization, C-terminal extension at the cytosolic side as well as oligomerization, and it finally culminates in the interaction with and gating of the plasma membrane Orai1 channel. The long CC1 helix of STIM1 (110 aa, Fig. 1*A*), situated between the ER transmembrane segment and CAD (aa 342–448), has been suggested to provide two interrelated functions as a segment that controls both C-terminal extension and bridging of the ER-PM distance (18, 27–29) of 11 to 14 nm for direct STIM1/Orai1 interaction (39). In an attempt to characterize the role of CC1 in controlling the STIM1 activation status at the cytosolic side, we compared the activity of the CAD domain in the absence as well as the presence of CC1. To mimic ER-targeted two-dimensional localization rather than using cytosolic expression, we engineered the CAD domain, with or without CC1, linked to the ER STIM1 transmembrane helix via a flexible, 32 glycine linker and visualized its cellular distribution by luminal attachment of the yellow fluorescent protein (Y), termed Y-TMG-OASF (Fig. 1*C*) or Y-TMG-CAD (Fig. 1*D*), respectively (see “Experimental Procedures”). Direct linkage of the fluorophore to the luminal side, without any regulatory (EF hand or SAM) domains present in wild-type STIM1, enabled focusing on the properties and potential interactions of STIM1 fragments secluded at the cytosolic side. Expression of Y-TMG-OASF, containing the CC1 prior to the CAD domain at the cytosolic side, displayed ER membrane localization similar to the control construct Y-TMG (Fig. 1*B*) lacking the cytosolic STIM1 portion. However, when the CC1 was omitted as in the Y-TMG-CAD construct, a distinctly different distribution pattern was obtained revealing clusters (Fig. 1*D*), likewise observable with STIM1-CAD (aa 1–448) following store depletion (40). Within CAD, such oligomerization might be mediated by the extended CC3 segment (CC3_449_: aa 388–449) (40, 41). Indeed, the expression of Y-TMG-CC3_449_ led to a similar, clustered distribution pattern (Fig. 1*E*), emphasizing its role in driving STIM1 higher-order oligomerization. To correspondingly assess the oligomerization propensity of this STIM region in biochemical experiments, we expressed and purified a recombinant CC3 fragment consisting of residues 388–491 (CC3ext). This fragment (>95% purity) yielded a single band on a reducing, Coomassie Blue-stained SDS-gel corresponding to the monomeric form with a molecular weight of 12.2 kDa (*inset*, Fig. 1*H*). The CC3ext fragment was highly α-helical based on far-UV circular dichroism (CD) spectra, which revealed negative ellipticity minima at ∼208 and 222 nm (Fig. 1*F*); further, the protein showed a cooperative unfolding profile in thermal melts with an apparent midpoint of temperature denaturation of ∼52.5 °C indicative of a well-folded polypeptide (Fig. 1*G*). Size exclusion chromatography (SEC) indicated that CC3ext forms higher order oligomers which elute in the void volume of the Superdex 200 gel filtration column (Fig. 1*H*); moreover, dilution of CC3ext did not alter the protein elution volume suggesting the oligomers were stably assembled (Fig. 1*H*). A C437A CC3ext mutant also formed higher order oligomers, ruling out a Cys-mediated assembly mechanism (not shown). Hence, the presence of the CC1 segment inhibits CC3_449_-mediated higher order oligomerization as well as cluster formation of CAD, a process that is tightly linked to the conformational rearrangement and activation status of STIM1 (33, 40). To validate the activation competence of the Y-TMG-CAD construct, also with respect to the missing CC1, we co-expressed it with CFP (C)-Orai1 to functionally address the specificity of the ER-PM spacer function, proposed for CC1, in whole-cell patch-clamp recordings. Y-TMG-CAD induced constitutively active, Ca^2+^ selective Orai1 currents that were sensitive to La^3+^ inhibition (Fig. 2*A*) and showed a reversal potential >40 mV (Fig. 2*B*), in line with the biophysical characteristics of a typical CRAC current (4, 13). The current density reached was in a similar range as that of Y-TMG-OASF (Fig. 2*A*) indicating that the absence of CC1 as a specific ER-PM spacer in Y-TMG-CAD did not interfere with its competence of fully activating Orai1 currents. As both Y-TMG-CAD as well as Y-TMG-OASF similarly co-clustered with Orai1 (Fig. 2, *C* and *D*), the active, extended form of Y-TMG-OASF together with the clustered distribution was induced by its interaction with Orai1 as recently reported (33). The Y-TMG-CC3_449_ did not exhibit co-localization with Orai1 (Fig. 2*E*) despite the clear clustered distribution, in line with the missing CC2 domain essential for the binding to the Orai1 C terminus (20, 35, 42).

Summarizing, these results demonstrate that the CC1 domain controls CAD exposure and cluster formation mediated via CC3_449_, but is dispensable as a specific spacer to bridge ER-PM distances for the activation of Orai1. As the CC1 segment is composed of three helices (see Fig. 1*A*), *i.e.* α_1_, α_2_, α_3_ with the latter also termed inhibitory helix (28), we analyzed in the following to which extent each helix controls the activation status of full-length STIM1.

##### Deletion of the First α-Helix in CC1 of STIM1 Results in Constitutive Coupling to and Full Activation of Orai1 Channels

**FIGURE 3.**
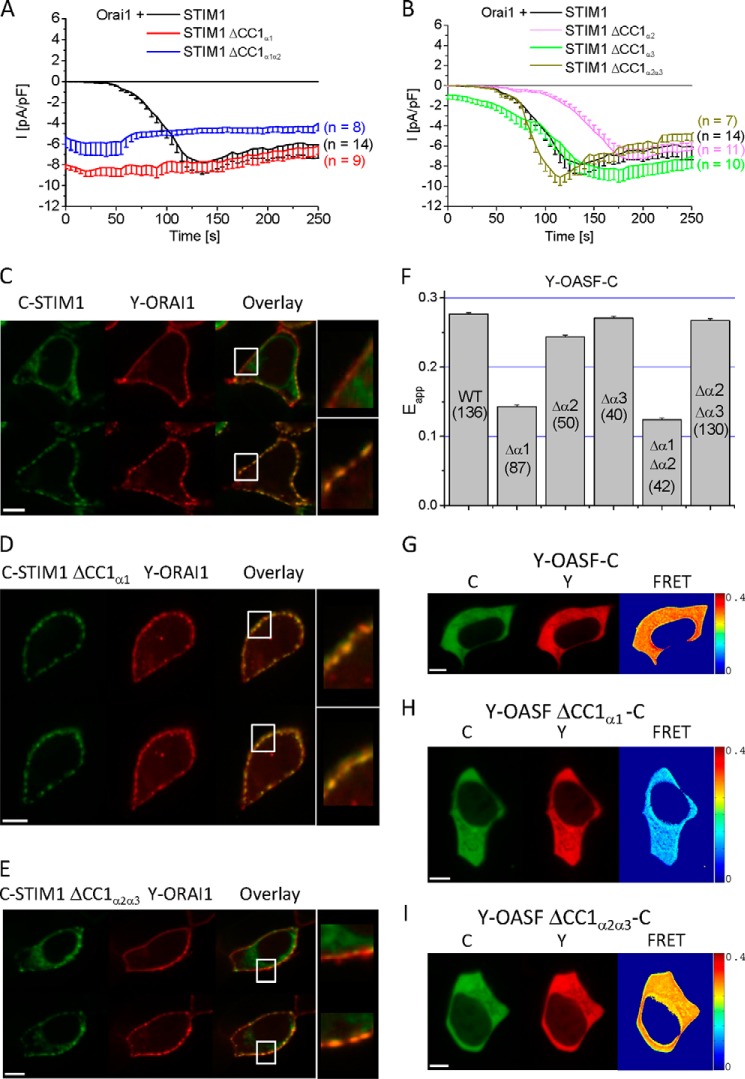
**Deletion of CC1_α1_ leads to STIM1 C-terminal extension and results in constitutive coupling to and full activation of Orai1 channels.** Time course of whole-cell inward currents from HEK293 cells co-expressing Orai1 together with (*A*) wild-type STIM1, STIM1 ΔCC1_α1_, and STIM1 ΔCC1_α1α2_ as well as (*B*) STIM1 ΔCC1_α2_, STIM1 ΔCC1_α3_ or STIM1 ΔCC1_α2α3_. The respective life cell image series (*C–E*) show localization and overlay under resting cell conditions (*upper panel*) and following 5 min store depletion with 2 μm TG in nominally free extracellular Ca^2+^ solutions (*lower panel*). *F*, block diagram summarizing FRET values (E_app_) of double-labeled OASF wild-type and deletion mutants (numbers in *brackets* indicate the quantity of cells measured): Y-OASF-C (WT), Y-OASF ΔCC1_α1_-C, Y-OASF ΔCC1_α2_-C, Y-OASF ΔCC1_α3_-C, Y-OASF ΔCC1_α1α2_-C, and Y-OASF ΔCC1_α2α3_-C. *G–I*, representative localization and calculated FRET image are shown for Y-OASF-C (WT), Y-OASF ΔCC1_α1_-C, and Y-OASF ΔCC1_α2α3_-C. Calibration bar is 5 μm throughout.

Based on structural predictions (Fig. 1*A*) (28, 35, 43), the three α helices of CC1 comprise the following segments (CC1_α1_: aa 238–271; CC1_α2_: aa 278–304 and CC1_α3_ (termed inhibitory helix, Ref. 28): aa 308–337). To systematically evaluate the impact of each α helix of CC1 on STIM1 co-clustering with and activation of Orai1 channels, we engineered various STIM1 deletion mutants (STIM1 ΔCC1_α1_, STIM1 ΔCC1_α2_, and STIM1 ΔCC1_α3_ as well as double helical deletions), with CC1_α1_ either deleted (Fig. 3*A*) or preserved (Fig. 3*B*). As a control, whole-cell patch-clamp recordings of HEK293 cells co-expressing wild-type STIM1 and Orai1 displayed the typical, store-dependent activation of CRAC currents evoked by passive store depletion (Fig. 3, *A* and *B*). In contrast, STIM1 ΔCC1_α1_ yielded constitutively and fully activated Orai1 currents, independent of store-depletion (Fig. 3*A*). Cells co-expressing STIM1 ΔCC1_α2α3_ and Orai1 (Fig. 3*B*) exhibited store-operated activation similar to wild-type STIM1, although STIM1 lacked the previously described, inhibitory CC1_α3_ helix together with CC1_α2_. The additional deletion of CC1_α2_ might have led to a more stabilized form of STIM1, as evident from initially reduced (ΔCC1_α2α3_) or delayed (ΔCC1_α2_) activation of Orai1 currents (Fig. 3*B*). Nonetheless, deletion of the CC1_α3_ helix alone, *i.e.* STIM1 ΔCC1_α3_, resulted only in small, constitutively active Orai1 currents that were further stimulated upon store-depletion to a similar level as obtained with wild-type STIM1 or STIM1 ΔCC1_α1_ (Fig. 3, *A* and *B*). In a quantitative comparison, the activation state of STIM1 ΔCC1_α3_ pre-activated only ∼10% of maximum Orai1 currents, similar as observed by Ref. 28, while >90% of activity was obtained with STIM1 ΔCC1_α1_ immediately after whole-cell break-in. These results point to a dominant role of CC1_α1_ over CC1_α3_ in controlling the activation status of STIM1 that is tightly linked to the extent of co-clustering with Orai1 (Fig. 3, *C–E*). Confocal fluorescence microscopy images visualized the typical co-clustering of co-expressed wild-type C-STIM1 and Y-Orai1 that was only observed following store-depletion by thapsigargin (Fig. 3*C*, *lower panel*), while in resting cells STIM1 localization was distinct to that of Orai1 (Fig. 3*C*, *upper panel*). In contrast, a store independent co-clustering of C-STIM1 ΔCC1_α1_ with Y-Orai1 was obtained (Fig. 3*D*, *upper and lower panels*), in line with constitutive, fully activated Orai1 currents (see Fig. 3*A*). Deletion of CC1_α2α3_ in STIM1 (Y-STIM1 ΔCC1_α2α3_) resulted, similar as with wild-type STIM1, only in clear co-clustering with Orai1 following store-depletion (Fig. 3*E*, *upper and lower panels*). Hence the degree of co-clustering of the various STIM1 mutants with Orai1 correlated with the extent of CRAC current activation demonstrating the most prominent impact of CC1_α1_ in controlling both functional correlates. In contrast to previous publications (27, 32, 33) reporting on an inhibitory function of CC1_α3_, our results indicate a predominant role of the CC1_α1_ helix in directing the activation status of STIM1 and the efficiency of CRAC current stimulation. As the activation of STIM1 involves a conformational C-terminal extension at the cytosolic side (30, 33, 34), we elucidated next the impact of each α-helix on such conformational transition.

##### The CC1_α1_ Helix Controls the Transition into an Extended STIM1 C-terminal Conformation

In an attempt to identify which of these α-helices exert control over STIM1 C-terminal conformational transitions, we utilized our recently developed OASF sensor construct that has enabled us to detect conformational rearrangements in the STIM1 C-terminal region (33). Specifically, the OASF fragment, consisting of CC1 and an extended CAD domain (comprising residues 233–474, see Fig. 1*A*) was double-labeled with Y/C (Y-OASF-C) at the N and C terminus, respectively, and the extent of FRET between these fluorophores was used as a reporter of conformational rearrangements (33). Wild-type Y-OASF-C resulted in the expected high FRET based on the close proximity of the N- and C-terminal fluorophores representing a tight conformation (Fig. 3, *F* and *G*) (33). Deletion of CC1_α1_ within the Y-OASF-C sensor (Y-OASF ΔCC1_α1_-C) led to a substantial reduction of FRET (Fig. 3, *F–H*), suggesting the transition to an extended conformation. Similarly, the deletion of both CC1_α1_ and CC1_α2_ with only the inhibitory CC1_α3_ helix remaining resulted in a decreased FRET suggesting an extended OASF conformation (Fig. 3*F*). Such an extended conformation is typically associated with the C-terminally activated form of STIM1 (30, 33, 34) in line with the observed constitutive coupling to and pronounced activation of Orai1 by STIM1 ΔCC1_α1_ and STIM1 ΔCC1_α1α2_ (Fig. 3*A*). Individual deletion of CC1_α2_ or CC1_α3_ only marginally decreased FRET (Fig. 3, *F* and *G*), pointing to minor C-terminal rearrangements. Moreover, even the concomitant deletion of CC1_α2_ and CC1_α3_ within the CC1 segment did not markedly reduce FRET (Fig. 3, *F* and *I*), suggesting the maintenance of a tight STIM1 C-terminal conformation consistent with the retained store-operated activation of CRAC currents (see Fig. 3*B*).

In summary, the complementary results of Fig. 3 obtained from electrophysiological, co-localization, and conformational sensor measurements pointed to a prominent role of the CC1_α1_ helix in controlling the activation status of STIM1 by locking its C terminus in a tight, quiescent conformation as long as the ER stores are full, possibly supported by CC1_α3_.

##### Homomerization Potential of Coiled-coil Domains as Well as Helical Segments

**FIGURE 4.**
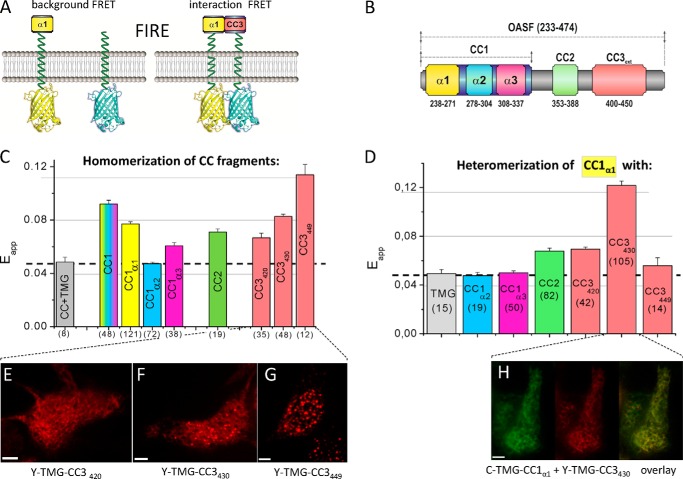
**Homomerization potential of coiled-coil domains as well as helical segments and their heteromerization with CC1_α1_.** *A*, FIRE system: *scheme* of FIRE constructs illustrating background FRET (control, *left*) and FRET derived from a specific interaction (*right*). *B*, scheme of human STIM1 OASF depicting CC1, CC2 and CC3 domains. The same color coding for respective domains is used in *C* and *D. C*, homomerization potential of individual CC or helical fragments (CC1_α1_ aa 233–276; CC1_α2_ aa 273–309, CC1_α3_ aa 303–342) determined by FIRE. *D*, *block diagram* depicting heteromeric interactions, obtained by FIRE, of CC1_α1_ with: TMG (control), CC1_α2_, CC1_α3_, CC2, CC3_420_, CC3_430_, and CC3_449_. *Dashed lines* in *C* and *D* represent the magnitude of the background signal. Number of cells studied are given in *brackets. E–G*, representative fluorescence images of Y-TMG-CC3 constructs of various lengths: (*E*) Y-TMG-CC3_420_ (no cluster formation), (*F*) Y-TMG-CC3_430_ (partial cluster formation), and (*G*) Y-TMG-CC3_449_ (strong cluster formation). *H*, representative fluorescence images of co-expressed Y-TMG-CC1_α1_ + Y-TMG-CC3_430_.revealing ER distribution without cluster formation. Calibration bar is 5 μm throughout.

The quiescent and activated state of the C-terminal STIM1, corresponding to a tight and extended conformation (34), might be mechanistically accomplished by intra- and/or intermolecular interactions of its CC and/or α-helical structures. Such interactions are expected to involve low-affinity binding sites for enabling transient conformational rearrangements. In an attempt to identify potential intra- and intermolecular domain interactions within the STIM1 C terminus, we utilized the established ER membrane-anchored TMG construct (Fig. 1) with individual CC or α-helical segments of STIM1 in a novel FRET approach termed FRET-derived Interaction in a Restricted Environment (FIRE). The luminal strand of these constructs, devoid of any regulatory domains, merely contains the respective fluorescent protein (C or Y) to allow for FRET-based measurements of two-dimensional interactions between ER-targeted, STIM1-derived fragments coupled via a 32 glycine long linker to the cytosolic side (Fig. 4*A*). FIRE enabled us to determine potential homomeric (Fig. 4*C*) as well as heteromeric (Fig. 4*D*) interactions between individual STIM1 segments (Fig. 4*B*), providing mechanistic clues about their involvement in STIM1 C-terminal rearrangements. The background of FIRE was determined from Y-TMG constructs with individually linked STIM1 fragments, each co-expressed with C-TMG and amounted to similar, small levels representing nonspecific collisional FRET (Fig. 4*C*, *dashed line*). Homomeric interactions were detected between CC1 domains yielding significant FRET (Fig. 4*C*), likely of weak affinity, as low-salt conditions have been required for their detection in co-immunoprecipitation experiments (40). In accordance, we failed to determine an increased homomeric FRET from cytosolically expressed C-/Y-labeled CC1 fragments (data not shown), underscoring the potential of FIRE in revealing weak interactions by mimicking the physiological two-dimensional ER environment. The contribution of each α-helix to the CC1 homomerization was evaluated next. FIRE revealed that CC1 homomerization was markedly mediated by CC1_α1_ and to a weaker extent by CC1_α3_, with CC1_α2_ lacking a significant homomerization potential (Fig. 4*C*), in support of a model where store depletion-induced luminal STIM1 di-/oligo-merization transpires through CC1 homomerization into a STIM1 C-terminal conformational extension (34). Further, we investigated the capability of CC2 and CC3 containing fragments to homomerically assemble as observed in our NMR structure and suggested by our higher-order assembly mechanism (35). Indeed, both CC2 (aa 344–399) and CC3 segments (aa 388–420/430/449) exhibited homomerization (Fig. 4*C*); interestingly, the extent of CC3-mediated interactions increased with increasing construct length. The CC3_449_ segment that fully included the STIM1 homomerization domain (SHD: aa 421–449) (22, 28, 41) revealed the highest FRET (Fig. 4*C*), in line with pronounced clustering and the formation of higher-order STIM1 oligomers as shown in Fig. 1, *E* and *H*, respectively. Deletion of the full SHD leading to the shortest CC3 segment analyzed (CC3_420_, aa 388–420) decreased FRET significantly (Fig. 4*C*) reaching the lowest level of homomerization consistent with substantial impairment of STIM1 homomerization (41). Indeed, the homo-/oligo-merization potential, visualized in confocal fluorescent images, revealed most pronounced clustering with CC3_449_, while increasing deletion of the SHD progressively reduced cluster formation (Fig. 4, *E–G*).

##### Heteromerization Potential of CC1_α1_ with STIM1 Segments

**FIGURE 5.**
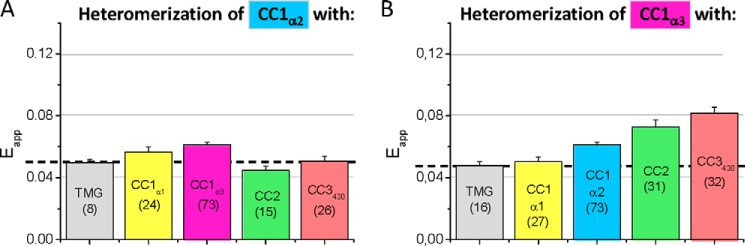
**Heteromeric interactions of the respective α_2_ and α_3_ helix of the CC1 domain with various OASF domains.** FRET (E_app_) determined from heteromeric interactions of (*A*) CC1 _α2_ and (*B*)CC1 _α3_ with various domains of OASF as depicted in Fig. 4. Interaction with TMG (no OASF fragment linked) represents control, with the *dashed lines* in *A* and *B* representing the magnitude of the background signal. Number of cells studied are given in *brackets*.

Next we focused on potential, heteromeric interactions of these STIM1 segments with the CC1_α1_ domain that might stabilize STIM1 in a tight, quiescent conformation and thus control its activation status. We tested the interaction potential of the CC1_α1_ domain with different STIM1 CC and α-helical structures using FIRE (Fig. 4*D*). While there were no heteromeric interactions detectable with the CC1_α2_ and the CC1_α3_ helices of CC1, a slight interaction was observed with CC2. Strikingly, the strongest interaction was obtained between CC1_α1_ and the CC3 containing intermediate segment (CC3_430_) (Fig. 3*D*). The CC1_α1_ interaction was substantially reduced both with the short (CC3_420_) and the longer (CC3_449_) CC3 segment. As the long CC3 segment included the full SHD (aa 420–449) (22, 41), its propensity for homomerization likely competed with the heteromeric interaction with the CC1_α1_ domain yielding the low FRET. The heteromeric interaction of the CC1_α1_ domain with the CC3 containing segment might control the activation state of STIM1 by preventing rearrangements and cluster formation. Indeed, co-expression of the CC1_α1_ and CC3_430_ domains that exhibited the strongest interaction as derived from FIRE, prevented formation of clusters (Fig. 4*H*), typically observed when CC3_430_ was expressed alone (see Fig. 4*F*). In addition we examined the interaction potential of CC1_α2_ or CC1_α3_ with CC2 or CC3 segments of CAD (Fig. 5). While CC1_α2_ did not clearly interact with any CC segment of the CAD domain (Fig. 5*A*), CC1_α3_ modestly coupled to CC2 and CC3 (Fig. 5*B*), consistent with the interactions derived from the crystal structure of *Caenorhabditis elegans* STIM1 (28) and the CC1_α3_-CC2 bundling observed in our recent solution structures (35).

In summary, the key heteromeric interaction between the CC1_α1_ domain and the CC3 segment might function as a coiled-coil clamp by locking STIM1 in a tight, quiescent conformation to prevent both CAD exposure and cluster formation. Weaker interactions were observed between CC1_α3_ (28) and CC2 as well as CC3 segments. Hence, our results suggest that the release of the interaction between the CC3_449_ segment and CC1_α1_ (supported by CC1_α3_) dominantly mediates the conformational switch from a tight into an extended STIM1 C terminus accompanied by CAD exposure, rearrangement, and cluster formation.

##### The CC1_α1_ Domain Is Sufficient for Controlling STIM1 Conformational Transition from a Quiescent to an Activated State

**FIGURE 6.**
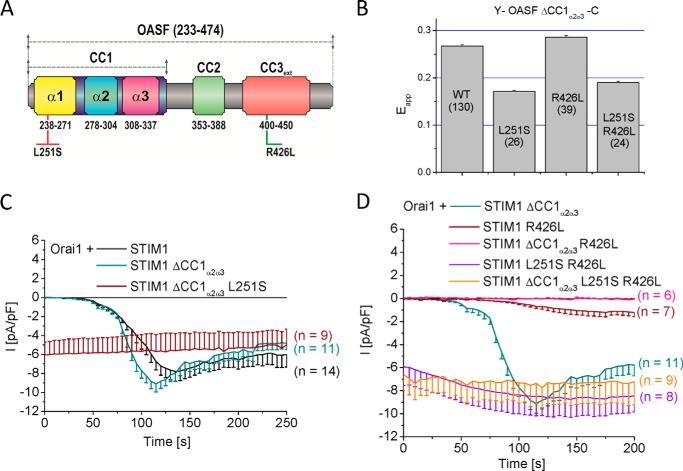
**The CC1_α1_ domain is sufficient for controlling STIM1 conformational transition from a quiescent to an activated state.** *A*, schematic diagram of human STIM1 OASF with highlighted point mutations that either weaken (*red* L251S) or enhance (*green* R426L) coiled-coil stability. *B*, *block diagram* summarizing FRET values (E_app_) of double-labeled Y-OASF ΔCC1_α2α3_-C (WT) as well as inserted point mutations (single L251S, single R426L, double L251S R426L). *C*, time courses of inward currents from whole-cell patch-clamp experiments co-expressing Orai1 with full-length STIM1 or STIM1 ΔCC1_α2α3_ or with the point mutation L251S introduced into STIM1 ΔCC1_α2α3_ that resulted in constitutively active inward currents. *D*, R426L mutation introduced either in wild type STIM1 or STIM1 ΔCC1_α2α3_ yielded highly attenuated inward Ca^2+^ currents when co-expressed with Orai1, while the additional point mutation L251S restored inward currents to similar levels in full length as well as STIM1 ΔCC1_α2α3_ L251S R426L mutants.

Our previous experiments suggest that the CC1_α1_ domain plays the key role in controlling STIM1 conformation, CAD exposure and clustering via its interaction with the CC3 containing segment. To underscore the predominant role of the CC1_α1_ domain, we generated STIM1 constructs with the CC1_α2_ and CC1_α3_ domains omitted. Additionally, to further support the concept that CC1_α1_ and CC3 interactions direct the formation of STIM1 C-terminal conformations, we utilized point mutations within CC1_α1_ (L251S) or CC3 (R426L) (Fig. 6*A*) that we have previously identified (33) for promoting the STIM1 C terminus extended or tight, respectively, conformation (33). With only the CC1_α1_ present, we analyzed its effect on the activation status of STIM1 or respective mutants utilizing both the OASF conformational sensor (Y-OASF-C, Fig. 6*B*) and the degree of Orai1 activation (Fig. 6, *C* and *D*) as read-out. Deletion of CC1_α2α3_ within the Y-OASF-C resulted in a high FRET suggesting a tight STIM1 C-terminal conformation, similar to the wild-type form (Fig. 6*B*). Introduction of the respective mutations in CC1_α1_ (L251S) or CC3 (R426L) that switched the wild-type Y-OASF-C into the extended or tight conformation induced similar conformational rearrangements in Y-OASF ΔCC1_α2α3_-C (Fig. 6*B*). Specifically, introducing L251S as well as L251S-R426L into Y-OASF ΔCC1_α2α3_-C drastically reduced FRET, while the R426L mutation slightly increased FRET. Electrophysiological recordings revealed clear, store-operated activation of Orai1 currents, evoked by passive store depletion, with the whole CC1 or only the CC1_α1_ domain present in full-length STIM1 (Fig. 6, *C* and *D*). Furthermore, point mutations that extended the conformation (L251S, L251S-R426L) introduced in STIM1 ΔCC1_α2α3_ yielded constitutively active Orai1 currents (Fig. 6, *C* and *D*). While the tightly packed STIM1 R426L mutant resulted in small store-operated currents (33), the sole presence of CC1_α1_ in the STIM1 ΔCC1_α2α3_ R426L mutant fully abrogated Orai1 current activation (Fig. 6*D*). These experiments clearly revealed that mutations within CC1_α1_ and CC3 helices correspondingly affected STIM1 C-terminal conformation as well as Orai1 activation, irrespective of whether only the CC1_α1_ domain or the whole CC1 domain was present. The double mutation, *i.e.* L251S in CC1_α1_ and R426L in CC3 additionally pointed to the predominant effect of CC1_α1_ on the STIM1 activation state superseding the stabilizing effect of mutated CC3. Thus, the main functional control of the activation status of STIM1 occurs via the CC1_α1_ helix.

##### Release of CC1_α1_-CC3 Interaction Is Required for Subsequent CC3_449_-mediated Cluster Formation

**FIGURE 7.**
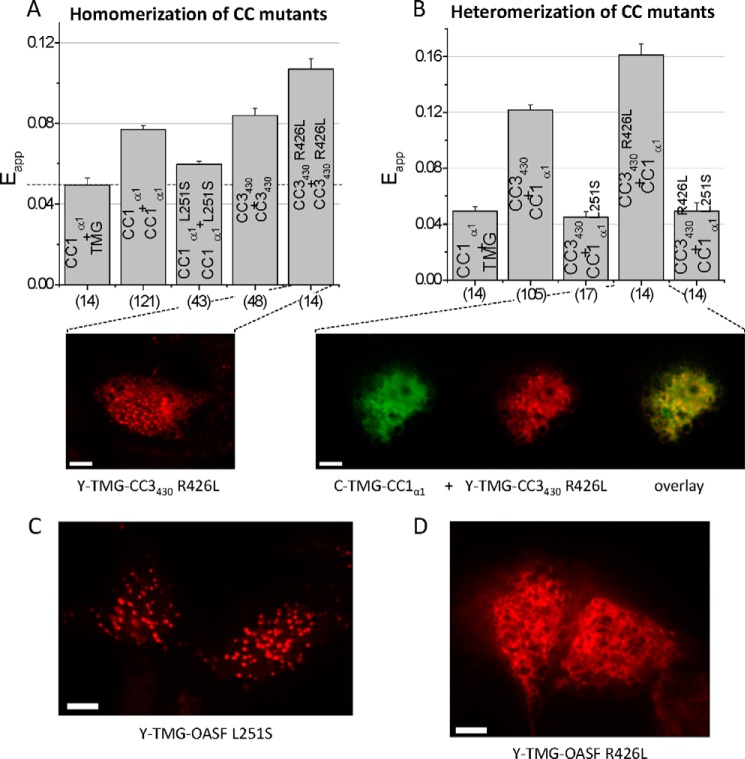
**Release of CC1_α1_-CC3 interaction is required for subsequent CC3_449_-mediated cluster formation.** Block diagrams summarizing the homomerization (*A*) as well as heteromerization potentials (*B*) of various CC or helical STIM1 fragments with individual point mutations as determined by FIRE. The *dashed line* in *A* represents the magnitude of the background signal. Fluorescence images from cells expressing (*A*, *lower panel*) Y-TMG-CC3_430_ R426L revealed cluster formation whereas cells co-expressing (*B*, *lower panel*) C-TMG-CC1_α1_ + Y-TMG-CC3_430_ R426L led to ER localization without cluster formation. *C*, representative fluorescence images from cell expressing Y-TMG-OASF L251S exhibited clear cluster formation while (*D*) Y-TMG-OASF R426L showed ER localization without clusters. Calibration bar is 5 μm throughout.

FIRE enabled us to directly address the impact of the above mutations on both homo- and heteromerization of mutated CC1_α1_ and CC3 fragments. While the L251S mutation decreased the moderate potential of CC1_α1_ to homomerize (Fig. 7*A*), it completely abolished the previously strong, heteromeric interaction with the CC3_430_ segment (Fig. 7*B*). These experiments showed that the heteromeric CC1_α1_-CC3 coupling was more drastically reduced by the L251S point mutations than the CC1_α1_-CC1_α1_ homomerization. Moreover, the observed CC1_α1_-CC3 uncoupling corresponded well with the extended conformation of the STIM1 C terminus (33, 34). On the contrary, the R426L mutation within the CC3 segment promoted the tight, quiescent STIM1 C-terminal conformation (Ref. 33 and see Fig. 6*B*). The individual impact of this mutation on CC3 homo- as well as heteromeric interaction with CC1_α1_,addressed by FIRE, revealed an increase in both CC3_430_ R426L homomeric (Fig. 7*A*) as well as heteromeric CC3_430_ R426L-CC1_α1_ interaction (Fig. 7*B*), in comparison to wild-type. Although the CC3_430_ homomerization was strengthened, the enhanced heteromeric CC1_α1_-CC3_430_ R426L interaction seemed to predominate and keep STIM1 in a quiescent, tight conformation. Indeed, while the sole expression of the CC3_430_ R426L resulted in the expected clustered distribution (Fig. 7*A*), its co-expression with the CC1_α1_ domain inhibited cluster formation (Fig. 7*B*). Hence, these experiments pointed to a sequential mechanism of STIM1 activation in as that the disruption of CC1_α1_-CC3 heteromeric coupling provides the initial, regulatory trigger for STIM1 transition into an extended conformation, which is accompanied by CAD exposure and subsequent CC3_449_-mediated formation of clusters. Accordingly, the ER-targeted Y-TMG-OASF L251S construct exhibited pronounced clustered structures (Fig. 7*C*), the formation of which was prevented in the Y-TMG-OASF R426L mutant form (Fig. 7*D*), as the enforced CC1_α1_-CC3 R426L interaction apparently inhibited the increased CC3-CC3 homomerization in these sequential activation steps by locking STIM1 in its tight conformation.

Combining the CC1_α1_ L251S mutant and the CC3 R426L segment, the individual mutations of which exerted opposing effects on heteromerization, revealed an abrogated CC1_α1_-CC3 interaction (Fig. 7*B*) pointing to a dominating effect of the former L251S mutation within the CC1_α1_-CC3 interaction interface. Indeed, the disrupted CC1_α1_-CC3 hetermomerization resulted in the extended C-terminal STIM1 conformation (Y-OASF L251S R426L-C; Fig. 6*B*) and revealed constitutively active currents for STIM1 L251S R426L as well as STIM1 ΔCC1_α2α3_ L251S R426L co-expressed with Orai1 (see Fig. 6*D*).

In summary, our data clearly demonstrate that the CC1_α1_-CC3 interaction is the key determinant for keeping STIM1 in a quiescent state. Upon store depletion, this clamp is released concomitant to a moderate CC1 homomerization and STIM1 conformational extension which is followed by CAD exposure and CC3_449_-mediated cluster formation essential for coupling to and activation of Orai1 channels. Mutations within CC1_α1_ or CC3 that abolish or strengthen this heteromeric interaction lock STIM1 into an extended or tight, respectively, conformation, resulting or failing in constitutively active Orai1 currents.

## DISCUSSION

Store-operated CRAC channel activation is mediated via direct coupling between Orai1 and the C terminus of STIM1 (13, 18, 19). The active conformation of STIM1 C terminus is acquired through CC1 dimerization that extends the cytosolic STIM1 (34), thereby exposing the SOAR/CAD domain for interaction with the Orai1 channel (33). While the CC1 domain might serve a spacer function between ER and PM (44), our data unequivocally show that the entire region is not necessary (CC1_α2_ and CC1_α3_ are dispensable) for bridging the ER-PM distance required for Orai activation. Rather, the primary role of the CC1 domain is to provide control over the release of the other STIM1 structural elements, which interact with the PM and Orai1. In this mechanism, the STIM1 C-terminal conformational extension is under predominant control of the CC1_α1_-CC3 interaction. The release of this coiled-coil clamp results in CAD exposure and CC3-mediated oligomerization as well as cluster formation, revealing dual levels of STIM1 auto-inihibition. The ability of CAD forming clusters has been first observed (40) with STIM1-CAD (aa 1–448), following store depletion. It has been suggested to stabilize STIM1 puncta formation, involving additionally a weak binding of the C-terminal polybasic STIM1 segment to plasma membrane PIP2 (34). The STIM1 C terminus key activation steps that involve mutual interactions between STIM1 CC domains were resolved here utilizing FIRE, where individual CC fragments or shorter helices were attached to the ER membrane mimicking the physiological two-dimensional environment. FIRE confirmed the interactions between CC1_α3_ and CC2 as well as CC3 observed in the high resolution solution (35) and crystal structures (28). More strikingly, however, previously unidentified interactions were revealed between CC1_α1_ and the CC2 as well as CC3 domain of STIM1. In electrophysiological experiments, deletion of CC1_α1_ in full-length STIM1 yielded constitutive Orai1 Ca^2+^ channels of maximum activity contrary to the much more moderate activation caused by CC1_α3_ deletion (28, 31). Accordingly, analogous deletion of CC1_α1_ or CC1_α3_ in the OASF conformational sensor indicated a substantial or minor, respectively, switch into the extended, active STIM1 form. Hence, FIRE revealed a novel CC1_α1_-CC3 interaction that predominantly controlled the overall activation status of STIM1. Apposition of the initial portions of the CC1 domain has been recently suggested to trigger the physical extension of STIM1 C terminus (34). In support, the whole CC1 domain as well as the CC1_α1_ helix exhibited moderate homomerization in FIRE mimicking two-dimensional environment, which was not detectable with analogous fragments cytosolically expressed (Ref. 34 and this study), probably due to transient affinity. The enforced CC1 homomerization in full-length STIM1 (34) elicited by association of the luminal domains following store depletion most likely results in the release of the dominant CC1_α1_-CC3 clamp, CAD exposure and helical rearrangements. Here we further demonstrated that the CC1_α1_-CC3 interface provided additional control over the formation of higher-order oligomers as well as clusters of CAD that were attributed to the robust oligomerization of the CC3 containing segment including the SHD domain (41). In line, cluster formation by the latter was abrogated when co-expressed with the CC1_α1_ domain.

Point mutations that modulated homo- and heteromeric coupling of CC1_α1_ and CC3 further supported the CC1_α1_-CC3 interaction as the dominant clamp directing conformational extension, as evaluated by FIRE. The L251S mutation within CC1_α1_ abolished its interaction with CC3, which, in the OASF moiety, yielded the extended conformation and resulted in the formation of clusters. Strikingly, this L251S mutation that abrogated the heteromeric CC1_α1_-CC3 interaction also partially reduced CC1_α1_ homomerization. Nonetheless, the activation status of OASF was controlled by the reduced CC1_α1_ L251S-CC3 interaction suggesting Leu-251 as a key molecular determinant of the CC1_α1_-CC3 interface.

Conversely, the R426L mutation within CC3 strengthened the heteromeric interaction with the CC1_α1_ domain and at the same time increased CC3 homomeric interaction, as revealed in FIRE. Despite the increased homomerization and robust clustering of the individual CC3 R426L domain, cluster formation was not apparent in the OASF R426L moiety underscoring the predominant role of the CC1_α1_-CC3 interaction in fixing the tight conformation and preventing cluster formation. Moreover, we suggest that the CC3 site mediating formation of higher-order oligomers as well as clustering (aa 421–449) overlaps with the CC1_α1_ interaction site, providing dual levels of STIM1 auto-inhibition through confinement of CAD and occupancy of CC3.

**FIGURE 8.**
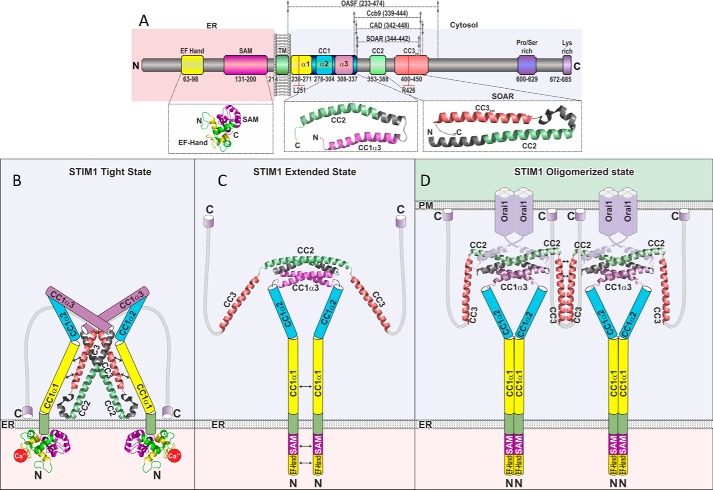
**Scheme depicting human STIM1 activation mechanism.** *A*, scheme of STIM1 depicting individual domains as well as positions L251 and R426, as denoted in Fig. 1*A. B–D*, hypothetical model reconciling the available key high resolution structural data for depicting structural changes and activation of human STIM1, following ER Ca^2+^ store depletion (*left to right*). *B*, in resting cells, the inactive quiescent form of STIM1 (STIM1 Tight State) is mainly accomplished via a coiled-coil clamp by heteromeric interaction between CC1_α1_ (*yellow*) and CC3 (*red*) resulting in a tight conformation. Residues at positions 251 and 426 are suggested as molecular determinants of the CC1_α1_-CC3 interface. We illustrated the V-shaped CAD/SOAR structure in a top-down position (Λ-shaped) facing the ER membrane with its positive charges. *C*, upon store depletion, a conformational change (STIM1 Extended State) is accomplished due to a release of the heteromeric CC1_α1_-CC3 clamp concomitant to a homomeric CC1_α1_-CC1_α1_ assembly, initiated by homomerization of the ER luminal part. *D*, finally, the STIM1 Oligomerized State is achieved via CC3 homomerization connecting STIM1 dimers in the oligomerized structure for coupling to and activation of Orai1 channels. For simplicity, STIM1 oligomerization is only depicted for two Orai1 dimers, with a suggested assembly of three Orai1 dimers with six STIM1 in a hexametric channel complex .

Based on functional and structural data of human STIM1, we present a model of human STIM1 activation, integrating the interaction of individual C-terminal domains, as identified by FIRE, into a sequential, molecular mechanism controlling the key steps of STIM1 C-terminal allostery driving CAD/SOAR exposure, helical rearrangement, and clustering (Fig. 8). The comparison of the interaction of individual domains and within the context of OASF, detected by their physiological, two-dimensional targeting to the ER membrane, indicates tight control of these activation steps by a predominant CC1_α1_-CC3 clamp. This clamp is supported by CC1_α3,_ yet the presence of CC1_α1_ is essential, in line with the STIM1 fragment (aa 315–462), which includes most of CC1_α3_ and is fully active (30). Combining our present data with the human SOAR/CAD (28) and our recent solution NMR structures (35) suggests a model where CC1 embraces the SOAR/CAD, via direct interactions between CC1_α1_ as well as CC1_α3_ with CC3/CC2 to stabilize the quiescent, tight conformation (Fig. 8*B*). Ca^2+^-depletion-dependent luminal EF-SAM domain destabilization and conformational changes at the secondary, tertiary, and quaternary structure levels provide the driving force, which initiates the conformational changes in the cytosolic STIM1 domains (10, 34, 45), promoting CC1_α1_ homo-merization (Ref. 40 and this work). This in turn triggers the release of the dominant CC1_α1_-CC3 clamp (Fig. 8*C*), accompanied by the extension of the C-terminal STIM1 conformation (28), and artificially promoted when CC1_α1_ is cross-linked (34). Once the CAD/SOAR region is released, a rearrangement of the CC2 and CC3 helices occurs, aligning CC2 helices for SOAP formation (35) and exposing CC3 for the higher-order oligomerization and clustering (Fig. 8*D*). The general feature of CAD/SOAR forming clusters demonstrates an avidity-driven (8) coupling mechanism to Orai1 in addition to an affinity-based interaction primarily achieved by the CC2 domain (20, 42). Although the wild-type CC1_α2_ does not markedly contribute to the CC1-CC3 clamp as shown here, a pathophysiological mutation therein (R304W) has been recently reported to result in constitutive, full STIM1 activity associated with the Stormorken syndrome (46, 47). It remains to be seen as to how this mutation affects the overall CC1 behavior within the context of OASF/STIM1, utilizing the here-developed FIRE as promising tool.

## Footnotes

4 The abbreviations used are:

ER: endoplasmic reticulum
CC: coiled-coil
FIRE: FRET-derived interaction in a restricted environment
STIM: stromal interaction molecule
SHD: STIM1 homomerization domain
PM: plasma membrane
Y: yellow fluorescent protein
C: cyan fluorescent protein
aa: amino acids.

## References

[1] Berridge M. J. Lipp P. Bootman M. D. (2000) Signal transduction. The calcium entry pas de deux. Science 287, 1604–1605 10733429 10.1126/science.287.5458.1604

[2] Liou J. Kim M. L. Heo W. D. Jones J. T. Myers J. W. Ferrell J. E. Jr. Meyer T. (2005) STIM is a Ca^2+^ sensor essential for Ca^2+^-store-depletion-triggered Ca^2+^ influx. Curr. Biol. 15, 1235–1241 16005298 10.1016/j.cub.2005.05.055 PMC3186072

[3] Roos J. DiGregorio P. J. Yeromin A. V. Ohlsen K. Lioudyno M. Zhang S. Safrina O. Kozak J. A. Wagner S. L. Cahalan M. D. Veliçelebi G. Stauderman K. A. (2005) STIM1, an essential and conserved component of store-operated Ca^2+^ channel function. J. Cell Biol. 169, 435–445 15866891 10.1083/jcb.200502019 PMC2171946

[4] Vig M. Peinelt C. Beck A. Koomoa D. L. Rabah D. Koblan-Huberson M. Kraft S. Turner H. Fleig A. Penner R. Kinet J. P. (2006) CRACM1 is a plasma membrane protein essential for store-operated Ca^2+^ entry. Science 312, 1220–1223 16645049 10.1126/science.1127883 PMC5685805

[5] Zhang S. L. Yu Y. Roos J. Kozak J. A. Deerinck T. J. Ellisman M. H. Stauderman K. A. Cahalan M. D. (2005) STIM1 is a Ca^2+^ sensor that activates CRAC channels and migrates from the Ca^2+^ store to the plasma membrane. Nature 437, 902–905 16208375 10.1038/nature04147 PMC1618826

[6] Yeromin A. V. Zhang S. L. Jiang W. Yu Y. Safrina O. Cahalan M. D. (2006) Molecular identification of the CRAC channel by altered ion selectivity in a mutant of Orai. Nature 443, 226–229 16921385 10.1038/nature05108 PMC2756048

[7] Feske S. Gwack Y. Prakriya M. Srikanth S. Puppel S. H. Tanasa B. Hogan P. G. Lewis R. S. Daly M. Rao A. (2006) A mutation in Orai1 causes immune deficiency by abrogating CRAC channel function. Nature 441, 179–185 16582901 10.1038/nature04702

[8] Luik R. M. Wang B. Prakriya M. Wu M. M. Lewis R. S. (2008) Oligomerization of STIM1 couples ER calcium depletion to CRAC channel activation. Nature 454, 538–542 18596693 10.1038/nature07065 PMC2712442

[9] Stathopulos P. B. Zheng L. Li G. Y. Plevin M. J. Ikura M. (2008) Structural and mechanistic insights into STIM1-mediated initiation of store-operated calcium entry. Cell 135, 110–122 18854159 10.1016/j.cell.2008.08.006

[10] Stathopulos P. B. Li G. Y. Plevin M. J. Ames J. B. Ikura M. (2006) Stored Ca2+ depletion-induced oligomerization of stromal interaction molecule 1 (STIM1) via the EF-SAM region: An initiation mechanism for capacitive Ca^2+^ entry. J. Biol. Chem. 281, 35855–35862 17020874 10.1074/jbc.M608247200

[11] Zheng L. Stathopulos P. B. Li G. Y. Ikura M. (2008) Biophysical characterization of the EF-hand and SAM domain containing Ca^2+^ sensory region of STIM1 and STIM2. Biochem. Biophys. Res. Commun. 369, 240–246 18166150 10.1016/j.bbrc.2007.12.129

[12] Wu M. M. Buchanan J. Luik R. M. Lewis R. S. (2006) Ca^2+^ store depletion causes STIM1 to accumulate in ER regions closely associated with the plasma membrane. J. Cell Biol. 174, 803–813 16966422 10.1083/jcb.200604014 PMC2064335

[13] Muik M. Frischauf I. Derler I. Fahrner M. Bergsmann J. Eder P. Schindl R. Hesch C. Polzinger B. Fritsch R. Kahr H. Madl J. Gruber H. Groschner K. Romanin C. (2008) Dynamic coupling of the putative coiled-coil domain of ORAI1 with STIM1 mediates ORAI1 channel activation. J. Biol. Chem. 283, 8014–8022 18187424 10.1074/jbc.M708898200

[14] Gwack Y. Srikanth S. Oh-Hora M. Hogan P. G. Lamperti E. D. Yamashita M. Gelinas C. Neems D. S. Sasaki Y. Feske S. Prakriya M. Rajewsky K. Rao A. (2008) Hair loss and defective T- and B-cell function in mice lacking ORAI1. Mol. Cell. Biol. 28, 5209–5222 18591248 10.1128/MCB.00360-08 PMC2519726

[15] Picard C. McCarl C. A. Papolos A. Khalil S. Lüthy K. Hivroz C. LeDeist F. Rieux-Laucat F. Rechavi G. Rao A. Fischer A. Feske S. (2009) STIM1 mutation associated with a syndrome of immunodeficiency and autoimmunity. N. Engl. J. Med. 360, 1971–1980 19420366 10.1056/NEJMoa0900082 PMC2851618

[16] Baba Y. Nishida K. Fujii Y. Hirano T. Hikida M. Kurosaki T. (2008) Essential function for the calcium sensor STIM1 in mast cell activation and anaphylactic responses. Nat. Immunol. 9, 81–88 18059272 10.1038/ni1546

[17] Zhang W. Halligan K. E. Zhang X. Bisaillon J. M. Gonzalez-Cobos J. C. Motiani R. K. Hu G. Vincent P. A. Zhou J. Barroso M. Singer H. A. Matrougui K. Trebak M. (2011) Orai1-mediated I (CRAC) is essential for neointima formation after vascular injury. Circ. Res. 109, 534–542 21737791 10.1161/CIRCRESAHA.111.246777 PMC3164514

[18] Park C. Y. Hoover P. J. Mullins F. M. Bachhawat P. Covington E. D. Raunser S. Walz T. Garcia K. C. Dolmetsch R. E. Lewis R. S. (2009) STIM1 clusters and activates CRAC channels via direct binding of a cytosolic domain to Orai1. Cell 136, 876–890 19249086 10.1016/j.cell.2009.02.014 PMC2670439

[19] Zhou Y. Meraner P. Kwon H. T. Machnes D. Oh-hora M. Zimmer J. Huang Y. Stura A. Rao A. Hogan P. G. (2010) STIM1 gates the store-operated calcium channel ORAI1 *in vitro*. Nat. Struct. Mol. Biol. 17, 112–116 20037597 10.1038/nsmb.1724 PMC2902271

[20] Frischauf I. Muik M. Derler I. Bergsmann J. Fahrner M. Schindl R. Groschner K. Romanin C. (2009) Molecular determinants of the coupling between STIM1 and Orai channels: differential activation of Orai1–3 channels by a STIM1 coiled-coil mutant. J. Biol. Chem. 284, 21696–21706 19506081 10.1074/jbc.M109.018408 PMC2755892

[21] Lis A. Zierler S. Peinelt C. Fleig A. Penner R. (2010) A single lysine in the N-terminal region of store-operated channels is critical for STIM1-mediated gating. J. Gen. Physiol. 136, 673–686 21115697 10.1085/jgp.201010484 PMC2995155

[22] Muik M. Schindl R. Fahrner M. Romanin C. (2012) Ca(2+) release-activated Ca(2+) (CRAC) current, structure, and function. Cell Mol. Life Sci. 69, 4163–4176 22802126 10.1007/s00018-012-1072-8 PMC3505497

[23] Schindl R. Muik M. Fahrner M. Derler I. Fritsch R. Bergsmann J. Romanin C. (2009) Recent progress on STIM1 domains controlling Orai activation. Cell Calcium 46, 227–232 19733393 10.1016/j.ceca.2009.08.003

[24] Engh A. Somasundaram A. Prakriya M. (2012) Permeation and gating mechanisms in store-operated CRAC channels. Front. Biosci. 17, 1613–1626 10.2741/4007 22201824

[25] Fahrner M. Derler I. Jardin I. Romanin C. (2013) The STIM1/Orai signaling machinery. Channels 7, 330–343 24107921 10.4161/chan.26742 PMC3913757

[26] Zheng L. Stathopulos P. B. Schindl R. Li G. Y. Romanin C. Ikura M. (2011) Auto-inhibitory role of the EF-SAM domain of STIM proteins in store-operated calcium entry. Proc. Natl. Acad. Sci. U. S. A. 108, 1337–1342 21217057 10.1073/pnas.1015125108 PMC3029719

[27] Yuan J. P. Zeng W. Dorwart M. R. Choi Y. J. Worley P. F. Muallem S. (2009) SOAR and the polybasic STIM1 domains gate and regulate Orai channels. Nat. Cell Biol. 11, 337–343 19182790 10.1038/ncb1842 PMC2663385

[28] Yang X. Jin H. Cai X. Li S. Shen Y. (2012) Structural and mechanistic insights into the activation of Stromal interaction molecule 1 (STIM1). Proc. Natl. Acad. Sci. U. S. A. 109, 5657–5662 22451904 10.1073/pnas.1118947109 PMC3326449

[29] Rothberg B. S. Wang Y. Gill D. L. (2013) Orai channel pore properties and gating by STIM: implications from the Orai crystal structure. Sci. Signal 6, pe9 23512988 10.1126/scisignal.2003971 PMC4746713

[30] Korzeniowski M. K. Manjarrés I. M. Varnai P. Balla T. (2010) Activation of STIM1-Orai1 involves an intramolecular switching mechanism. Sci. Signal 3, ra82 21081754 10.1126/scisignal.2001122 PMC3408607

[31] Yu F. Sun L. Hubrack S. Selvaraj S. Machaca K. (2013) Intramolecular shielding maintains the ER Ca(2+) sensor STIM1 in an inactive conformation. J. Cell Sci. 126, 2401–2410 23572507 10.1242/jcs.117200

[32] Yu J. Zhang H. Zhang M. Deng Y. Wang H. Lu J. Xu T. Xu P. (2013) An aromatic amino acid in the coiled-coil 1 domain plays a crucial role in the auto-inhibitory mechanism of STIM1. Biochem. J. 454, 401–409 23795811 10.1042/BJ20130292

[33] Muik M. Fahrner M. Schindl R. Stathopulos P. Frischauf I. Derler I. Plenk P. Lackner B. Groschner K. Ikura M. Romanin C. (2011) STIM1 couples to ORAI1 via an intramolecular transition into an extended conformation. EMBO J. 30, 1678–1689 21427704 10.1038/emboj.2011.79 PMC3101990

[34] Zhou Y. Srinivasan P. Razavi S. Seymour S. Meraner P. Gudlur A. Stathopulos P. B. Ikura M. Rao A. Hogan P. G. (2013) Initial activation of STIM1, the regulator of store-operated calcium entry. Nat. Struct. Mol. Biol. 20, 973–981 23851458 10.1038/nsmb.2625 PMC3784406

[35] Stathopulos P. B. Schindl R. Fahrner M. Zheng L. Gasmi-Seabrook G. M. Muik M. Romanin C. Ikura M. (2013) STIM1/Orai1 coiled-coil interplay in the regulation of store-operated calcium entry. Nature Commun. 4, 2963 24351972 10.1038/ncomms3963 PMC3927877

[36] Hou X. Pedi L. Diver M. M. Long S. B. (2012) Crystal structure of the calcium release-activated calcium channel Orai. Science 338, 1308–1313 23180775 10.1126/science.1228757 PMC3695727

[37] Derler I. Hofbauer M. Kahr H. Fritsch R. Muik M. Kepplinger K. Hack M. E. Moritz S. Schindl R. Groschner K. Romanin C. (2006) Dynamic but not constitutive association of calmodulin with rat TRPV6 channels enables fine tuning of Ca^2+^-dependent inactivation. J. Physiol. 577, 31–44 16959851 10.1113/jphysiol.2006.118661 PMC2000671

[38] Zal T. Gascoigne N. R. (2004) Photobleaching-corrected FRET efficiency imaging of live cells. Biophys. J. 86, 3923–3939 15189889 10.1529/biophysj.103.022087 PMC1304294

[39] Várnai P. Tóth B. Tóth D. J. Hunyady L. Balla T. (2007) Visualization and manipulation of plasma membrane-endoplasmic reticulum contact sites indicates the presence of additional molecular components within the STIM1-Orai1 Complex. J. Biol. Chem. 282, 29678–29690 17684017 10.1074/jbc.M704339200

[40] Covington E. D. Wu M. M. Lewis R. S. (2010) Essential role for the CRAC activation domain in store-dependent oligomerization of STIM1. Mol. Biol. Cell 21, 1897–1907 20375143 10.1091/mbc.E10-02-0145 PMC2877647

[41] Muik M. Fahrner M. Derler I. Schindl R. Bergsmann J. Frischauf I. Groschner K. Romanin C. (2009) A Cytosolic Homomerization and a Modulatory Domain within STIM1 C Terminus Determine Coupling to ORAI1 Channels. J. Biol. Chem. 284, 8421–8426 19189966 10.1074/jbc.C800229200 PMC2659200

[42] Wang X. Wang Y. Zhou Y. Hendron E. Mancarella S. Andrake M. D. Rothberg B. S. Soboloff J. Gill D. L. (2014) Distinct Orai-coupling domains in STIM1 and STIM2 define the Orai-activating site. Nat. Commun. 5, 3183 24492416 10.1038/ncomms4183 PMC3995141

[43] Soboloff J. Rothberg B. S. Madesh M. Gill D. L. (2012) STIM proteins: dynamic calcium signal transducers. Nat. Rev. Mol. Cell Biol. 13, 549–565 22914293 10.1038/nrm3414 PMC3458427

[44] Hogan P. G. Lewis R. S. Rao A. (2010) Molecular basis of calcium signaling in lymphocytes: STIM and ORAI. Annu. Rev. Immunol. 28, 491–533 20307213 10.1146/annurev.immunol.021908.132550 PMC2861828

[45] Furukawa Y. Teraguchi S. Ikegami T. Dagliyan O. Jin L. Hall D. Dokholyan N. V. Namba K. Akira S. Kurosaki T. Baba Y. Standley D. M. (2014) Intrinsic disorder mediates cooperative signal transduction in STIM1. J. Mol. Biol. 426, 2082–2097 24650897 10.1016/j.jmb.2014.03.006

[46] Misceo D. Holmgren A. Louch W. E. Holme P. A. Mizobuchi M. Morales R. J. De Paula A. M. Stray-Pedersen A. Lyle R. Dalhus B. Christensen G. Stormorken H. Tjønnfjord G. E. Frengen E. (2014) A Dominant STIM1 Mutation Causes Stormorken Syndrome. Hum. Mutat. 35, 556–564 24619930 10.1002/humu.22544

[47] Nesin V. Wiley G. Kousi M. Ong E. C. Lehmann T. Nicholl D. J. Suri M. Shahrizaila N. Katsanis N. Gaffney P. M. Wierenga K. J. Tsiokas L. (2014) Activating mutations in STIM1 and ORAI1 cause overlapping syndromes of tubular myopathy and congenital miosis. Proc. Natl. Acad. Sci. U. S. A. 111, 4197–4202 24591628 10.1073/pnas.1312520111 PMC3964084

[48] Singh A. Hamedinger D. Hoda J. C. Gebhart M. Koschak A. Romanin C. Striessnig J. (2006) C-terminal modulator controls Ca^2+^-dependent gating of Ca(v)1.4 L-type Ca^2+^ channels. Nat. Neurosci. 9, 1108–1116 16921373 10.1038/nn1751

